# The stable microbiome of inter and sub-tidal anemone species under increasing *p*CO_2_

**DOI:** 10.1038/srep37387

**Published:** 2016-11-23

**Authors:** Erinn M. Muller, Maoz Fine, Kim B. Ritchie

**Affiliations:** 1 Mote Marine Laboratory, Sarasota, FL 34236, USA; 2 The Mina and Everard Goodman Faculty of Life Sciences, Bar-Ilan University, Ramat-Gan 52900, Israel; 3 The Interuniversity Institute of Marine Science in Eilat, P.O.B. 469 Eilat 88103, Israel; 4 The University of South Carolina, Beaufort SC 29902, USA.

## Abstract

Increasing levels of *p*CO_2_ within the oceans will select for resistant organisms such as anemones, which may thrive under ocean acidification conditions. However, increasing *p*CO_2_ may alter the bacterial community of marine organisms, significantly affecting the health status of the host. A pH gradient associated with a natural volcanic vent system within Levante Bay, Vulcano Island, Italy, was used to test the effects of ocean acidification on the bacterial community of two anemone species *in situ, Anemonia viridis* and *Actinia equina* using 16 S rDNA pyrosequencing. Results showed the bacterial community of the two anemone species differed significantly from each other primarily because of differences in the Gammaproteobacteria and Epsilonproteobacteria abundances. The bacterial communities did not differ within species among sites with decreasing pH except for *A. viridis* at the vent site (pH = 6.05). In addition to low pH, the vent site contains trace metals and sulfide that may have influenced the bacteria community of *A. viridis*. The stability of the bacterial community from pH 8.1 to pH 7.4, coupled with previous experiments showing the lack of, or beneficial changes within anemones living under low pH conditions indicates that *A. viridis* and *A. equina* will be winners under future ocean acidification scenarios.

Ocean acidification, or an increase in oceanic *p*CO_2_, may have significant effects on organisms within the marine environment. The increase in oceanic *p*CO_2_ could profoundly affect organismal physiology, the carbonate structure of the substrate, and overall ecosystem functioning^1^,^2^. Under this changing environment certain species may thrive while others may suffer negative consequences. Identifying the winners and losers of changing oceanic *p*CO_2_ will provide insight into future shifts in community assemblages within the marine environment.

While ocean acidification may cause reduced rates of survival, growth, calcification and reproduction on several different calcifying species, from corals to coccolithophores^3^, non-calcifying organisms, especially ones that photosynthesize, may be positively affected by ocean acidification. Therefore, reduced oceanic pH may be beneficial for some organisms like anemones^3^,^4^,^5^. For example, prolonged exposure to high *p*CO_2_ showed an increase in the growth of the algal symbiont within the anemone *Anthopleura elegantissima*^6^.

*Anemonia viridis* is a symbiotic sea anemone found within the northeastern Atlantic and Mediterranean Sea and is often used as a model for cnidarian studies^7^. Interestingly, *A. viridis* has a high capacity to buffer intracellular pH conditions even when exposed to low pH seawater^8^ suggesting that overall host physiology may not be severely impacted by ocean acidification. Several studies have been conducted on *A. viridis* in natural volcanic CO_2_ seeps, specifically off the coast of Vulcano, (Aeolian Islands) Italy. At this location Suggett *et al*.^4^ showed that *A. viridis* increased productivity (measured as gross photosynthesis:respiration ratios) with increasing *p*CO_2_ along a natural gradient, suggesting that this particular anemone may thrive under future *p*CO_2_ scenarios. Within the same site, Horwitz *et al*.^5^ showed that autotrophy of *A. viridis* increased with increasing *p*CO_2_, which illustrates the overall flexibility of this anemone species. Interestingly, *A. viridis* in sites with varying levels of *p*CO_2_ showed no significant differences in chlorophyll *a* concentrations, *Symbiodinium* densities or protein concentrations although the *Symbiodinium* mitotic index within anemones increased under high *p*CO_2_^4^,^5^. In light of these previous physiological studies, *A. viridis* appears to be either resistant to or positively affected by ocean acidification and may be considered a “winner” under acidified conditions^4^.

In concordance with changes in the physiological state of *A. viridis*, ocean acidification conditions may alter the microbial communities of this anemone. Indeed, physiological changes of the host because of declining pH may lead to altered states within the bacterial communities. Marine microbes respond rapidly to environmental shifts^9^, are key components of the oceanic nutrient cycles^10^, and are important members of the host microbiome, providing essential functions for host health^11^. Some studies indicate that microbial communities will not be altered by ocean acidification conditions^12^,^13^. For example, Oliver *et al*.^13^ showed that there was no change in community abundance, structure or composition of the bacterial fraction of microbial plankton under ocean acidification conditions. Bacterial communities associated with oxic sediments, however, appear to significantly differ at sites with naturally low pH compared with control sites^14^,^15^. Furthermore, other studies indicate that microbial communities associated with host organisms may in fact change under elevated *p*CO2. Webster *et al*.^16^ documented differences in microbial communities associated with the coral species *Acropora millipora*, the foraminifera *Marginopora vertebralis*, the crustose coralline algae *Hydrolithon onkodes*, and reef biofilms between treatments of pH 8.1 and 7.9 conditions. Alternatively, a more recent study by Webster *et al*.^17^ showed that the coral species *Acropora millipora* and *Seriatopora hystrix*, the foraminifera *Marginopora vertebralis* and *Heterostegina depressa*, the CCA *Hydrolithon onkodes* and the sea urchin *Echinometra* sp. showed no difference in bacterial community composition between pH 8.1 and 7.9 treatments, and only detected differences in the bacterial communities when temperature treatments were high and pH was low^17^. A study of *A. millipora* and *Porites cylindrica* at a natural CO_2_ seep in Papua New Guinea, however, indicated differences in the bacterial communities of these corals at the low pH site, predominantly caused by a loss of the bacterial symbiont *Endozoicomonas*^18^. Analysis of the bacteria associated with *A. viridis* from Ischia Island (Gulf of Naples), Italy showed that the microbial communities of this species were more diverse in samples from a low pH site (7.0) compared with samples from a reference site (8.1), but there was no difference in antibiotic activity of associated bacteria when comparing the two sites^19^. The lack of consistency and preliminary nature of the research testing the effects of ocean acidification on bacterial communities indicates a significant knowledge gap in need of study.

*Actinia equina* is a non-photosymbiotic sea anemone commonly found in the rocky intertidal of the North Atlantic Ocean and the Mediterranean Sea^7^. This species of anemone can survive for prolonged periods of time out of the water and are even found covered in sand^20^. Along the pH gradient in Levante Bay, *A. equina* is very common but is exposed to high *p*CO_2_ conditions only when fully submerged, while during low tide it is often exposed to atmospheric CO_2_ levels. The continuous diurnal exposure to a variable environment may suggest that this species is more resistant to environmental changes than others^21^. A study on the lysozyme activity of *A. equina* mucus, however, showed a peak in activity at pH 6^22^. These results suggest that one of the best defenses against bacterial invasion, activity of the enzyme lysozyme, will increase for *A. equina* under reduced pH, thereby protecting the resident microbiome of the anemone leading to overall maintenance in host health. How the resident microbiome, including the bacterial communities of *A. equina*, will be impacted by ocean acidification is unknown.

The objectives of the present study were to i) characterize and compare the microbial communities of two anemone species with different life history strategies found along a natural pH gradient within the sublittoral zone of Levante Bay, Vulcano Island, Italy (*A. viridis*, a subtidal species with an endosymbiont, and *A. equina,* an intertidal species with no endosymbiont), and ii) to determine whether the bacterial assemblages of these two species differed with decreasing levels of pH.

## Methods

### Sample collection

**Figure 1:**
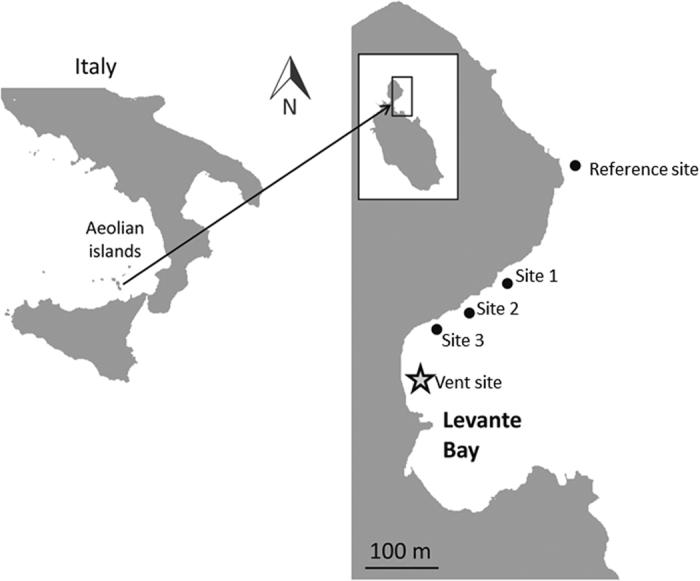
Map of sites sampled along the shoreline of Levante Bay, Vulcano Island Italy. Figure modified from Horwitz *et al*.^5^.

Anemone samples were collected on June 21, 2013 between 10:00 and 11:00 hrs at sites along the sublittoral between 0.25 and 2 m depth in Levante Bay, North Vulcano Island (38° 25′N, 14° 57′E). Water temperature was 23 °C and did not vary within the sampled area. Samples were taken along the gradient from five sites: the reference site (500 m northeast of the vent; pH 8.15), site 1 (390 m from the vent, pH 8.03), site 2 (300 m from the vent, pH 7.86), site 3 (240 m from the vent, pH 7.44), and the vent site (15 m from the vent; pH 6.05; Fig. 1). Although extensive physical parameters were not collected simultaneously with the anemone samples, thorough geochemical surveys have been conducted previously that characterize Levante Bay spatially and temporally^23^,^24^,^25^. These surveys indicate that sites 1, 2, 3 and the reference site differ little except for within their carbonate chemistry composition (see Boatta *et al*.^23^ and Johnson *et al*.^24^ for further details in the carbonate chemistry within this area). Temperature, salinity, total alkalinity, sulfur concentrations, nitrate, phosphate, sediment texture, and total nitrogen and total organic carbon content within the sediment do not significantly differ along the north shore of Levante Bay^14^,^23^,^24^,^25^. However, nitrite and silicate have shown significant differences between site 3 and the reference site location^25^. The vent site, in addition to having extremely low pH conditions (6.05), also contains elevated levels of trace elements, iron, and hydrogen sulfide compared with other sites further from the CO_2_ vent^23^,^26^. Anemone samples for the present study included ~300 μg of tentacles from three randomly selected individuals of *A. viridis* at each site, and ~300 μg of both tentacles and the body column from three randomly selected *A. equina*. Samples were collected with sterile scissors by collectors wearing sterile nitrile gloves. Once the anemone sample was collected it was removed from the water and placed within a sterile 10 ml polystyrene tube. Samples of *A. equina* were not collected from the vent site because this species was absent within this location. All samples were stored in 10 ml of RNA Later (Qiagen, Inc), cryopreserved using liquid nitrogen within 1 hour of collection^27^, and transported back to Mote Marine Laboratory (Sarasota FL) in a liquid nitrogen dry shipper for subsequent analyses.

### Next Generation Sequencing

Source DNA was extracted from each anemone sample using the MoBio Powersoil DNA isolation kit with a modified protocol (MoBio Inc., Carlsbad, CA). The microbiome of each sample was analyzed using 16 S rDNA 454 pyrosequencing. A modified amplicon pyrosequencing (bTEFAP) procedure was performed with 16 S universal Eubacterial primers, a modified 27 F and 519 R primer. Polymerase chain reaction was carried out using a single-step 30 cycle HotStarTaq Plus Master Mix Kit (Qiagen, Valencia, CA) under the following conditions: 94 °C for 3 minutes, 28 cycles of 94 °C for 30 seconds; 53 °C for 40 seconds and 72 °C for 1 minute with a final elongation step at 72 °C for 5 minutes. Amplicon products from different samples were combined equally and purified using Agencourt Ampure beads (Agencourt Bioscience Corporation, MA, USA). Samples were sequenced via Roche 454 FLX titanium instruments and reagents following manufacturer’s guidelines. The sequence data was processed at MRDNA laboratory (www.mrdnalab.com, Shallowater, TX) using a standardized analysis pipeline developed and implemented by MRDNA laboratory. Briefly, sequences were depleted of barcodes and primers and then short sequences <200 bp were removed. Sequences with ambiguous base calls as well as sequences with homopolymer runs exceeding 6 bp were removed. Sequences were then de-noised and chimeras were removed. Operational taxonomic units (OTUs) were defined after removal of singleton sequences, clustering at 3% divergence (97% similarity)^28^. Final OTUs were taxonomically classified using BLASTn against a curated database derived from GreenGenes, RDPII and NCBI (www.ncbi.nlm.nih.gov; http://rdp.cme.msu.edu)^29^ and compiled into each taxonomic level (Supplementary Table 1).

The sequencing data from this study were submitted to GenBank within the National Center for Biotechnology Information (http://www.ncbi.nlm.nih.gov) under Accession numbers KX149130 – KX150371 for 16 S rRNA gene pyrosequencing.

### Statistics

Rarefaction curves, which estimated the coverage of species richness, were created from the average number of reads within each bacteria species detected using pyrosequencing for each anemone species. Coverage, the probability of occurrence of the bacterial species observed in the sample within each site, was calculated using the Zhang-Huang estimator from the ‘entropart’ package in R^30^,^31^,^32^. The Shannon, Simpson, inverse Simpson diversity indices, and Pielou’s richness of the bacterial community were calculated at the species taxonomic level for each sample. Differences in diversity or richness among sites were tested for each anemone species using analysis of variance (ANOVA), after passing assumptions of normality and homoscedasticity. However, a Kruskal Wallis test was used for the Simpson and inverse Simpson index of the *A. viridis* data because of non-normal distributions within the dataset. The percent composition of bacterial groups from each sample was also analyzed at the OTU level using a factorial permutation multivariate analysis of variance (PERMANOVA) with anemone species and sites designated as the two independent variables. PERMANOVA was also used to determine if there were differences among sites within species. Pairwise PERMANOVAs were used to determine which sites where different between and within anemone species when significant differences were detected within the omnibus test. A similarity percentages (SIMPER) analysis within the ‘vegan’ package of the statistical program R^33^ was used to provide a relative dissimilarity index among sites and also provided the percent dissimilarity caused by each bacterial OTU. The top five contributors to dissimilarity were tested for differences among sites using Kruskal Wallis tests. Dunn’s test with a Bonferroni correction was used for posthoc comparisons when significant differences occurred using the ‘dunn.test’ package in R^34^. Bacterial OTU data were then processed through non-metric multidimensional scaling (NMDS), which applied the rank orders of data to represent the position of communities in multidimensional space using a reduced number of dimensions that can be easily plotted and visualized. The NMDS results were then plotted in two-dimensional ordination space using the ‘vegan’ package in R^33^. The relative abundance of bacterial classes and the relative abundances of genera within major classes were also plotted for visualization. A heatmap of the most common genera, those found in >1% abundance, was created using the R packages ‘Heatplus’^35^, ‘gplots’^36^, ‘RColorBrewer’^37^ and ‘vegan’^33^.

## Results

No visual difference in size, color or health was observed in *Anemonia viridis* or *Actinia equina* individuals from different sites. Both species were plentiful along the shoreline of Levante Bay and appeared robust regardless of the location. There was however, no *A. equina* within the vent site due to a lack of intertidal substrate and habitat.

### Metadata from pyrosequencing

Pyrosequencing resulted in an average 21,530 (±2,339) sequence reads per *A. viridis* sample, and 21,367 (±5,160) reads per *A. equina* sample (see GenBank accession numbers for sequencing information). For *A. viridis*, the average base pair length detected for each read was 444 (±4) bp and average GC content was 57% (±0.1). For *A. equina*, the average base pair length per sequence was 459 (±2) bp and average GC content was 57% (±0.1). Prior to analyses, the data were standardized using a subset of 6,167 random sequence reads per replicate sample, the lowest coverage found within a sample after sequencing.

### Rarefaction curve

The rarefaction curve from the *A. viridis* samples indicated that a substantial portion of the bacterial species were detected within our dataset (Supplementary Figure 1). Coverage analyses indicated that >99% of the bacterial species within each site were detected even after subsetting samples to standardize for sequencing reads (Supplementary Table 2).

### Bacterial diversity

**Figure 2:**
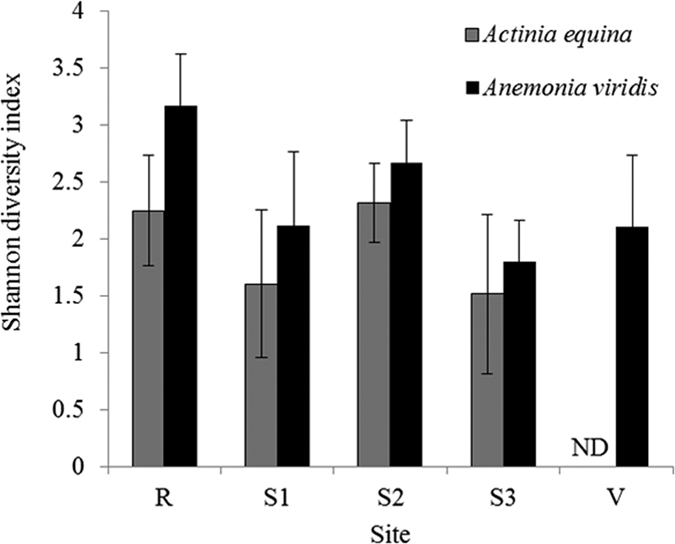
Shannon diversity index of bacterial species found within samples of *Anemonia viridis* and *Actinia equina* along the pH gradient of Levante Bay, Vulcano, Italy (R = reference site, S1 = site 1, S2 = site 2, S3 = site 3, V = vent site). ND represents no data because *A. equina* was not found within the vent site.

Statistical analyses showed no difference in diversity or richness within the bacterial species among sites for either *A. viridis* or *A. equina* (Supplementary Table 3; Fig. 2). Overall, diversity was lower in site 1 and site 3 for both *A. viridis* and *A. equina*.

### Comparison between anemone species

**Figure 3:**
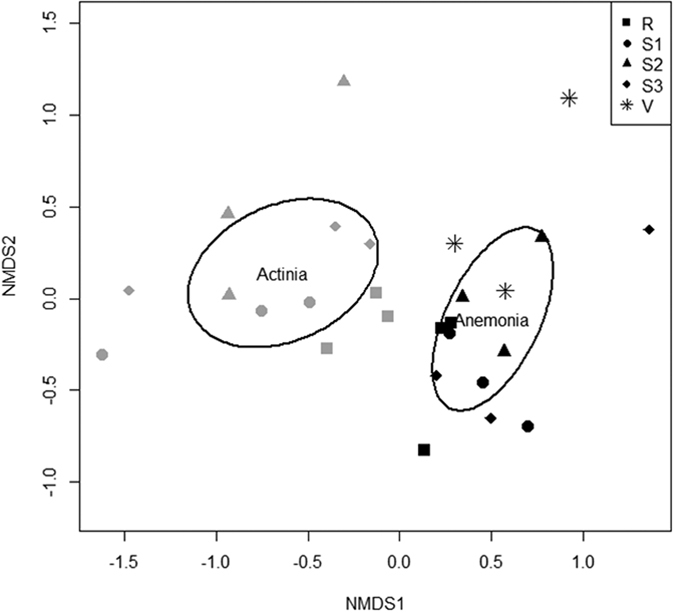
NMDS Ordination plot of the bacterial communities of *Anemonia viridis* and *Actinia equina* (stress = 0.18). Black symbols represent samples of *A. viridis*, whereas grey symbols represent samples of *A. equina*. Open black ovals represent 95% confidence intervals of the NMDS analysis.

**Figure 4:**
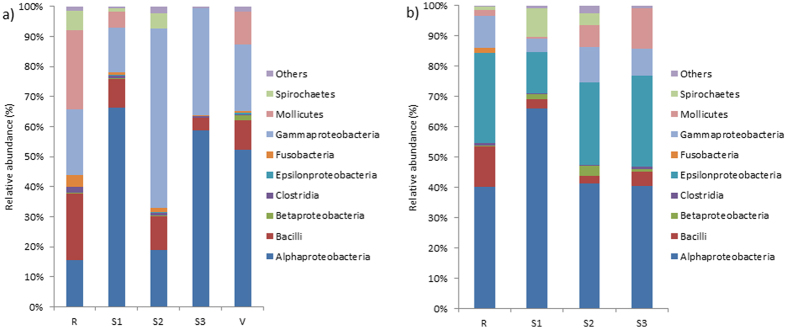
Relative abundances of bacterial classes present in (**a**) *Anemonia viridis* and (**b**) *Actinia equina* collected at different sites along a pH gradient in Levante Bay, Vulcano, Italy (R = reference site, S1 = site 1, S2 = site 2, S3 = site 3, V = vent site). Bacteria present in <3% relative abundance are grouped within ‘others’.

The bacterial community of *A. viridis* significantly differed from the bacterial community of *A. equina* (F(1,25) = 5.798, R2: 0.188, p < 0.001). The NMDS ordination plot indicates little to no overlap in the relative abundance of bacteria from these two anemone species (Fig. 3). Comparisons of the bacterial class composition showed that there were significantly more Gammaproteobacteria (*X*^2^(1) = 9.152, p = 0.003) and significantly less Epsilonproteobacteria (*X*^2^(1) = 17.626, p < 0.001) within *A. viridis* compared with *A. equina*. Overall, *A. viridis* had a relative bacterial abundance of 27.69% (±6.4% SE) Gammaproteobacteria, whereas *A. equina* contained only 5.26% (±1.18%) Gammaproteobacteria (Fig. 4). *A. viridis* had on average only 0.17% (±0.07%) Epsilonproteobacteria, whereas *A. equina* had on average 19.13% (±6.77%) Epsilonproteobacteria (Fig. 4). Importantly, a significant difference remained between species even when the vent site data of *A. viridis* was removed from the comparison (F(1,22) = 5.848, R2: 0.210, p < 0.001). The factorial PERMANOVA also showed that when using both anemone species data there were no differences detected among sites, nor was there an interaction effect between sites and anemone species (sites: F(4,18) = 1.359, R2 = 0.168, p = 0.065; species x site: F(3,18) = 0.964, R2 = 0.089, p = 0.523).

### Bacterial community analysis within anemone species

**Figure 5:**
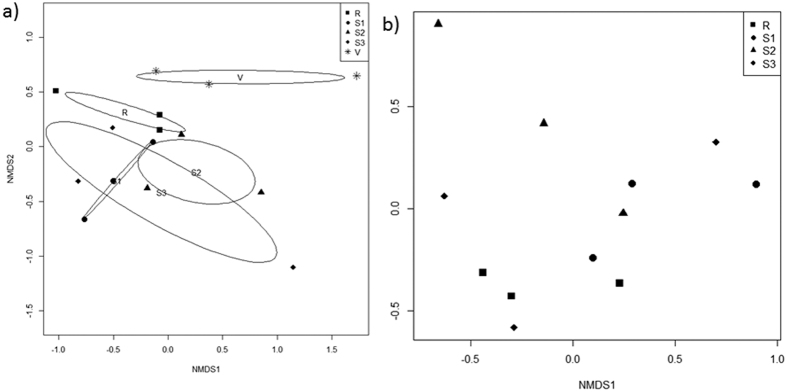
NMDS ordination plots of bacterial community composition by OTUs for (**a**) *Anemonia viridis* (stress = 0.13; n = 3 per site, R = reference site, S1 = site 1, S2 = site 2, S3 = site 3, V = vent site) and (**b**) *Actinia equina* (stress = 0.11; n = 3 per site, R = reference site, S1 = site 1, S2 = site 2, S3 = site 3). Open black ovals represent 95% confidence intervals of the NMDS analysis.

**Figure 6:**
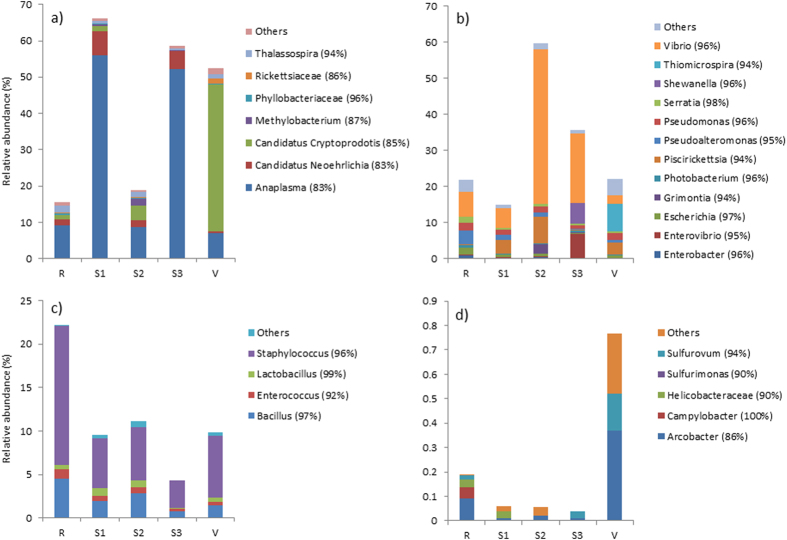
Relative abundance of (**a**) Alphaproteobacteria, (**b**) Gammaproteobacteria, (**c**) Bacilli, and (**d**) Epsilonproteobacteria present in *Anemonia viridis.* Parentheses after the Genus name indicate percent homology of identification (R = reference site, S1 = site 1, S2 = site 2, S3 = site 3, V = vent site). Bacteria present in <3% relative abundance are grouped within ‘others’.

There was a significant difference in bacterial communities of *A. viridis* among sites (F(4,10) = 1.47; R2: 0.370, p = 0.046; Fig. 5a) when this anemone species was analyzed independently. Pairwise PERMANOVAs, however, showed no significant differences between sites tested likely because of high levels of variation within sites (Supplementary Table 4). The heatmap created of the most dominant genera visually displays the high levels of variation among the samples, little convergence within sites, and the dominance by a few bacterial genera, mostly within the Alphaprotebacteria class (Supplementary Figure 2). SIMPER analysis of the Bray Curtis dissimilarity index (Table 1) showed that the most dissimilar site was the vent site compared with all other sites sampled and Site 3 compared with the reference site. The NMDS ordination plot of the *A. viridis* data illustrates the separation of the vent site compared with all other sites (Fig. 5a). The SIMPER analysis also showed that differences between the vent site and all other sites were primarily driven by five OTUs: 1) an OTU with 85% similarity to *Candidatus Cryptoprodotis* (Fig. 6a), 2) an OTU with 83% similarity to the genus *Anaplasma* (Fig. 6a), 3) *Spiroplasma* spp (homology identified as 83%; data not shown), 4) *Vibrio* (homology identified as 92%; Fig. 6b), and 5) *Thiomicrospira* (homology identified as 94%; Fig. 6b). Comparisons of these genera among sites using nonparametric Kruskal-Wallis tests showed significant differences for the OTU similar to *Candidatus Cryptoprodotis* (*X*^*2*^(4) = 9.43, p = 0.049; Supplementary Figure 3), for *Spiroplasma* (*X*^2^ (4) = 12.897, p = 0.012; Supplementary Figure 3), and also for *Thiomicrospira* (*X*^2^ (4) = 13.796, p = 0.008; Supplementary Figure 3). The posthoc Dunn’s test with a Bonferroni correction showed the OTU similar to *Candidatus Cryptoprodotis* was significantly higher at the vent site compared with site 3 (Z = −2.647, p = 0.041; Fig. 6a; Supplementary Figure 3). However, there were no differences detected in *Spiroplasma* spp. among sites using this conservative posthoc test (Supplementary Figure 3). *Thiomicrospira* was significantly more prevalent at the vent site compared with all other sites (Z = −2.936, p = 0.017; Fig. 6b; Supplementary Figure 3).

There were no significant differences in the bacterial communities of *A. equina* among sites (PERMANOVA F(3,8) = 0.844, R2 = 0.245, p = 0.627; Fig. 5b). Furthermore, the SIMPER dissimilarity index (Table 1) showed less dissimilarity among sites in the *A. equina* samples when compared with the *A. viridis* samples.

### Relative bacterial abundance

The relative abundance of bacterial classes in *A. viridis* showed variations among sites, although there were no significant differences detected among sites for any of the dominant bacterial classes (Fig. 4a; Supplementary Table 5). Qualitative trends among bacterial classes and the genera that dominate those classes are reported. Alphaproteobacteria showed reduced abundance in the reference site as well as site 2, as compared with other sites (Fig. 4a). When examining the Alphaproteobacteria separately, the relative abundance of this class was dominated by an OTU 83% similar to *Anaplasma* in the reference site, as well as sites 1, 2, and 3 (Fig. 6a). However, the dominant Alphaproteobacteria at the vent site was the OTU 85% similar to *Candidatus Cryptoprodotis.* As noted previously, the Dunn’s posthoc test showed that the vent site had a significantly higher abundance of this OTU, when compared with site 3 (Z = −2.647, p = 0.041; Supplementary Figure 3). At the reference site there was a noticeable increase in *Mollicutes* (Fig. 4a), which was dominated by the genus *Spiroplasma* (Supplementary Figure 3). Gammaproteobacteria was the most common bacterial class in sites 2 and 3 (Fig. 4a). When qualitatively examining this class separately, the shift in Gammaproteobacteria was driven by an increase in *Vibrio* at these two sites (Fig. 6b), although no significance was detected statistically. The bacterial class Bacilli, which were dominated by *Staphylococcus* (Fig. 6c) had the highest relative abundance in the reference site samples, but were present in decreased levels at all other sites. Although Epsilonproteobacteria was generally rare in *A. viridis* (Fig. 4a) this class was dominated by chemolithoautotrophs that oxidize sulfur and showed a marked increase in the vent site compared with all other sites (Fig. 6d; data not statistically significant).

**Figure 7:**
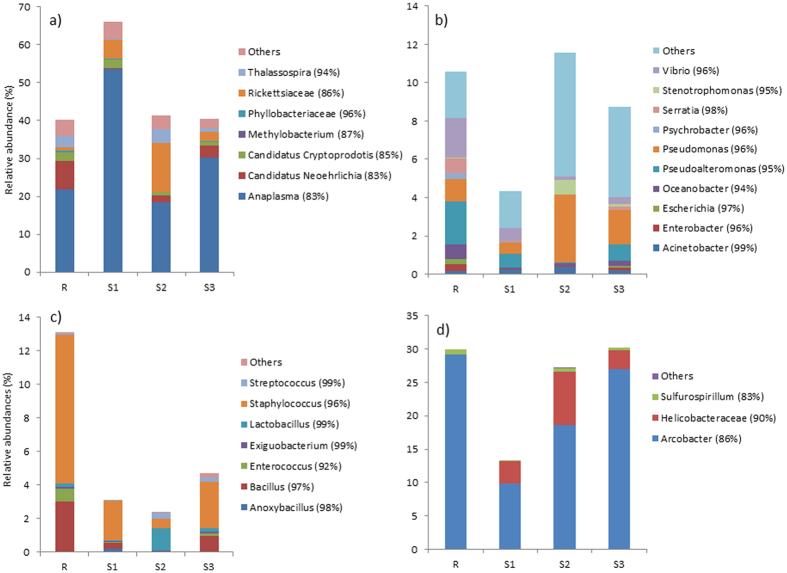
Relative abundance of (**a**) Alphaproteobacteria, (**b**) Gammaproteobacteria, (**c**) Bacilli, (**d**) Epsiolonproteobacteria present in *Actinia equina.* Parentheses after the Genus name indicate percent homology of identification (R = reference site, S1 = site 1, S2 = site 2, S3 = site 3). Bacteria present in <3% relative abundance are grouped within ‘others’.

There were no significant differences among sites detected for the most dominant bacterial classes found within *A. equina* (Supplementary Table 6). Qualitative trends among bacterial classes and the genera that dominate those classes are reported. The relative abundance of bacterial classes of *A. equina* showed that Alphaproteobacteria was one of the most common classes of bacteria in the samples (Fig. 4b). Alphaproteobacteria abundances were highest at site 1 compared with all other sites, although this change was not statistically significant. The increase in relative abundance for Alphaproteobacteria at site 1 was a result of higher abundances of the OTU 83% similar to *Anaplasma* (Fig. 7a). Qualitatively, the relative amount of *Rickettsiaceae* within the Alphaproteobacteria class increased in site 2 compared with the other sites. Gammaproteobacteria remained relatively stable among sites (Fig. 7b), but the relative amount of *Pseudomonas* within this class increased substantially at sites closest to the vent (Fig. 7b). Mollicutes, dominated by the genus *Mycoplasma* (data not shown) tended to increase as pH decreased (i.e., at sites closest to the vent; Fig. 4b). Bacilli, which was dominated by *Staphylococcus* (Fig. 7c) was highest in the reference site compared with all of the other sites (Fig. 7c). Interestingly, Epsilonproteobacteria was the second most common class within *Actinia* (Fig. 7d); *Arcobacter* dominated this class (Fig. 7d).

## Discussion

The bacterial composition of the two anemone species, *A. viridis* and *A. equina*, was significantly different from each other, which may have been driven by variations in interspecific traits between host genera or because of differences in the tissues sampled between *A. viridis* and *A. equina*. Samples of *A. viridis* contained only tentacles, whereas *A. equina* samples included part of the body column because tentacles within this species are extremely small. Therefore, the difference in bacterial assemblages between the species may also be because *A. equina* samples contained tentacles, body wall, and potentially gonads and guts. Previous studies indicate that bacterial aggregates may be specific to certain tissue types within anemones. For example, *Vibrio*-like aggregates appear to congregate on the tentacles and oral disc of *Aiptasia pallida*^38^, whereas bacterial aggregates in *Metridium senile* were found on the tentacles^39^. Furthermore, another study showed bacterial aggregates only within the mesenteries of *Nematostella vectensis*^40^. Future studies should focus on whether differences detected within the present study were from host species specificity, tissue type, or both factors.

While both anemone species showed an abundance of Alphaproteobacteria, there were significantly more Gammaproteobacteria within *A. viridis* compared with *A. equina. A. viridis* is known to harbor *Symbiodinium* of clade A19^41^ as a symbiotic dinoflagellate partner, whereas *A. equina* does not harbor dinoflagellate endosymbionts. Bourne *et al*.^42^ showed that invertebrates containing photosynthetic symbionts often have more Gammaproteobacteria, many of which produce dimethylsulfoniopropionate (DMSP). Additionally, Ritchie^43^ suggests there are obligate symbioses between *Symbiodinium* and bacteria within the Alphaproteobacterial class, such as *Ruegeria* spp. and *Roseobacteriales* spp. Alphaproteobacteria were a dominant component of the *A. viridis* bacterial assemblage, further exemplifying the relationship between photosymbiosis and anemone-bacterial communities. Furthermore, the lack of *Symbiodinium* may require that *A. equina* host a more diverse assemblage of bacterial functional groups.

An additional difference in bacterial communities detected between these two species of anemones was driven by the relative abundances of the Epsilonproteobacteria class. Epsilonproteobacteria were heavily abundant in all *A. equina* samples, even those from the reference site. However, these bacteria were minimally present in *A. viridis*. The Epsilonproteobacteria within both anemones were primarily composed of sulfide oxidizing bacteria such as *Sulfurimonas, Helicobacter, Sulfurovum*, and *Sulfospirillum*. Epsilonproteobacteria such as these have been found in hydrothermal vents off of Okinawa and deep vents off the northeast Pacific Ocean, with optimal growth rates at approximately pH 6.5^44^,^45^. Boatta *et al*.^23^ showed that at sites along the north shore of Levante Bay (>300 m from the vent) sulfide was absent and sulfate levels were typical for oceanic waters. The results of the present study, however, suggest that there may be some sulfide present within the seawater along the northern shore of Levante Bay that is influencing the bacterial community of the anemones. However, the low abundance of Epsilonproteobacteria within *A. viridis* and the high abundance of this class within *A. equina* may suggest that other factors may be affecting the microbial community. *A. equina* is often exposed to the atmospheric conditions during low tide, which may influence the bacterial assemblages found associated with this species. Atmospheric gas composition is known to affect microbial growth and abundance, knowledge that has been utilized for extending the shelf-life of perishable foods by slowing bacterial growth^46^. Since *A. equina* is exposed to air often during low tides, bacterial communities may be shaped by the atmospheric environment as well as the seawater. *Arcobacter* was also a large component of the Epsilonproteobacteria community of *A. equina. Acrobacter* are often pathogenic bacteria and aerotolerant, likely an important trait for inhabiting an intertidal anemone that is exposed to air during low tide.

There was a significant difference detected within the bacterial communities of *A. viridis* among sites. However, all sites showed similar bacterial communities except for samples from the vent site. Community-based analyses on the OTU data showed no difference in the bacterial assemblages when comparing the reference site, site 1, site 2, and site 3 even though the pH within these sites ranged from 8.15 to 7.44. Additionally, statistical differences detected within the genus level showed changes only within the vent sites. This lack of change within all other sampled sites indicates a high level of stability in the microbiome of *A. viridis*. The bacterial community of the vent site, pH of 6.05, distinctly separated from the other sites in the NMDS plot and SIMPER analysis showed that the vent site was the most different from all other sites sampled. In 2100 the pH of the oceans is predicted to decline to 7.7^47^, a value much higher than that within site 3 (7.44), and substantially higher than the vent site (6.05). Differences that were detected within the vent site appeared to be particularly driven by the changes in composition within the Alphaproteobacteria and Gammaproteobacteria classes. Within the Alphaproteobacteria class there was an increase in the abundance of the OTU 85% similar to *Candidatus Cryptoprodotis* within the vent site*. Candidatus Cryptoprodotis* is a Rickettsia-like bacterium that has been associated with the ciliate species *Pseudomicrothorax dubius*^48^. Rickettsia-like organisms are obligate intracellular gram-negative bacterial parasites associated with a variety of vertebrate and invertebrate hosts^48^. Low homology within this particular OTU, however, suggests a previously undescribed genus.

The Gammaproteobacteria in *A. viridis* showed high diversity within sites, but mainly consisted of *Vibrio* spp, a known marine pathogen. This bacterium, however, did not show a trend with changing pH and increased at site 2 compared with all other sites. The vent site showed a different relative abundance of bacteria within the Gammaproteobacteria group with less *Vibrio* present and a high relative abundance of *Thiomicrospira*, an obligate chemolithoautotrophic sulfur oxidizer^39^. Again, the presence of *Thiomicrospira* is likely directly related to the high amount of hydrogen sulfide present within the vent site^23^,^49^,^50^,^51^.

Although there were no differences in the bacterial community among sites with pH values above 6.05, the vent site did harbor a significantly different bacterial community within A. viridis. The difference in the bacterial community of *A. viridis* at the vent site could be attributed to additional different characteristics found within that location. Extremely low pH conditions of 6.05 consistently recorded within this site may have significantly altered the microbial communities. Variations in *p*CO_2_ resulted in significantly different microbial communities of biofilms grown on glass slides^52^ as well as the microphytobenthic assemblage within Levante Bay^25^. Indeed, environmental influences shape microbial communities and drive spatial variation of microbial diversity^53^. Perhaps within *A. viridis* there is a very low pH threshold that was exceeded within the vent site, which led to altered microbial communities at this location. Alternatively, the presence of elevated trace elements may have influenced the bacterial community of the anemones at the vent site. Horwitz *et al*.^26^ showed there were differences in trace elements associated with the environment near the vent with higher levels of trace metals such as Fe, Mn, and Cr. Additionally, Fe, Cu, Pb, Co, and Sr increased in both the pedal disk and tentacles of *A. viridis* when trace elements were high^26^. Mn, Fe, Co, Ni, Zn, Cu, and Cd are considered trace elements essential for the proper function of marine microorganisms^54^. Indeed, these trace elements and their bio-availability may be particularly influential in changing the bacterial communities of *A. viridis* at the vent site. Further studies that isolate the influence of *p*CO_2_ from these trace elements are needed to completely understand the contribution each may play in controlling bacterial abundances on anemones.

Microbes living within host macroorganisms such as anemones may also be influenced by intrinsic factors. At the vent site, under high *p*CO_2_, *A. viridis* has significantly greater levels of autotrophy^5^. Suggett *et al*.^4^ also showed higher levels of photosynthesis and respiration in *A. viridis* under high *p*CO_2_ conditions compared with control sites. Increased levels of photosynthesis may also lead to elevated levels of photoassimilates available to bacterial communities, causing shifts at the community level. Studies of the bacterial communities within the rhizosphere of plants showed increasing sugars led to significant changes within the assemblage^55^. Additionally, saltwater mesocosm studies showed the addition of glucose led to significant changes in the bacterial community, specifically within the Alphaproteobacteria and Gammaproteobacteria classes^56^. Borell *et al*.^41^ also showed a decrease in dimethylsulfoniopropionate (DMSP) as well as superoxide dismutase (SOD) in *A. viridis* at sites within Levante Bay that had elevated *p*CO_2_ compared with the control site. Marine bacteria, such as Roseobacters found within the Alphaproteobacteria class, consume DMSP and catabolize DMSP to DMS, the main natural source of reduced sulfur released to the atmosphere^57^,^58^,^59^. These additional changes to the intrinsic composition of the host anemone and bioavailability of DMSP for catabolism, may in turn affect the bacterial community found within *A. viridis*. It is critical to note, however, that most studies found these physiological differences within the pH range of 8.2 to 7.44. The present study showed no differences in the bacterial assemblages of *A. viridis* within this range, and found that only within an extreme environment, such as the vent site, was there significant changes within the microbiome of this tolerant anemone species.

Spatial differences of the bacterial community within *A. viridis* must also be considered when interpreting the results of the present study. Meron *et al*.^19^ characterized the bacterial groups of *A. viridis* of Ischia Island, Italy, utilizing another natural CO_2_ vent. Interestingly, the bacterial groups that dominated the Ischia samples showed different relative abundances compared with the present samples from a nearby location. In Ischia, samples at the ambient (8.1) pH site were dominated by Firmicutes and Gammaproteobacteria. The present Vulcano samples did not pick up any OTUs within the Firmicute class. Alphaproteobacteria composed <10% of the bacteria present in Ischia, whereas our samples showed between 10 and 50% relative abundance of this bacterial group. Diversity also increased for samples under a pH of 7 in Ischia, whereas the Vulcano samples showed no significant change in diversity among sites tested. Differences in the bacterial communities between these two relatively close locations indicate that local factors causing different oceanographic characteristics are significantly influencing the bacterial communities of the *A. viridis* between these two locations.

There were no significant differences detected in the bacterial communities of *A. equina* among the different sites. The lack of change in bacterial community composition of the sampled sites for *A. equina*, suggests that this species, like *A. viridis,* may be tolerant of large variations in *p*CO_2_. Since *A. equina* is an intertidal species, bacteria associated with this anemone may be additionally tolerant to recurrent variations in temperature, irradiance, and salinity between tides, which could be reflected in the microbial group stability. The high relative abundance of sulfur-oxidizing bacteria, which characteristically have high growth rates at low pH conditions^44^,^45^ also, suggests an innate tolerance of the bacterial communities to low pH conditions. Site 3 regularly has a pH value of 7.44, levels much lower than predicted even 100 years from now for the oceans^47^, yet the bacterial communities of these *A. equina* samples were not different than those found at pH of 8.1. The results of the present study suggest that the bacterial community of *A. equina* may be unchanged as pH continues to decrease, and suggest a high tolerance of the *A. equina* holobiont to ocean acidification conditions.

The present study showed that there were high levels of bacterial community stability within the anemones sampled along a CO_2_ gradient in Levante Bay, Italy. Differences between species, however, demonstrate the significance the host plays in structuring the bacterial community, a function that is maintained throughout the pH gradient. The ambient conditions in Levante Bay are formed by a combination of the incoming seawater driven by the tides and weather conditions, and the vent emissions, which includes CO_2_ and trace metals. The lack of a difference of the bacterial communities in all sites other than the vent site, again indicate substantial resilience of community structure for these two anemones as *p*CO_2_ increases. In fact, the lack of a change in the bacterial community among all other sites tested suggests that these assemblages are resilient to changes in pH alone. Little difference in bacterial communities among sites outside of the vent also indicates that similar dynamics are influencing the bacterial communities even though pH is significantly different among these sites. Previous studies show that ocean acidification significantly changes the bacterial assemblages found in biofilms^52^ and within the sediments^14^ of Levante Bay. The high stability of the microbial community of both anemones tested in the present study indicates that the host itself may buffer the impact of ocean acidification on the anemones’ microbiome. Changes within the host physiology along the *p*CO_2_ gradient also did not influence bacterial communities within the host. Therefore, living within the tissues of the anemone offers stability and independence from ambient conditions at least until extreme thresholds are reached such as at the vent site. Scleractinian corals contain mechanisms within the fluid at the tissue-skeleton interface that mitigates the effects of ocean acidification^60^. The host anemones within the present study also provide a protective environment for the bacterial assemblages perhaps through the mitigation on intracellular pH^8^. Within the host, the bacteria are provided nutrition, a hospitable environment, and potentially protection from changes within the ambient environment. These results emphasize the resilience of anemones at both the macro- and micro-scale, under changing *p*CO_2_ conditions and indicate that *A. viridis* and *A. equina* will be winners under future oceanic conditions.

## Additional Information

**Accession Codes**: Accession file SUB1478661: KX149130 - KX150371.

**How to cite this article**: Muller, E. M. *et al*. The stable microbiome of inter and sub-tidal anemone species under increasing *p*CO_2_. *Sci. Rep.* **6**, 37387; doi: 10.1038/srep37387 (2016).

**Publisher's note:** Springer Nature remains neutral with regard to jurisdictional claims in published maps and institutional affiliations.

## Supplementary Material

Supplementary Information

## Figures and Tables

**Table 1:** Dissimilarity values, which could range from 0 (least dissimilar) to 1 (most dissimilar) of the bacterial communities of *Anemonia viridis* and *Actinia equina* between sites calculated using SIMPER.

*Anemonia viridis*		*Actinia equina*	
Site comparison	Dissimilarity value	Site comparison	Dissimilarity value
V vs S3	0.903	R vs S2	0.804
V vs S1	0.855	S2 vs S3	0.784
R vs S3	0.854	R vs S3	0.745
V vs S2	0.844	S1 vs S3	0.734
V vs R	0.835	R vs S1	0.724
S2 vs S3	0.830	S1 vs S2	0.716
R vs S2	0.785		
R vs S1	0.769		
S1 vs S2	0.760		
S1 vs S3	0.684		
