# Supplementary material for: The stable microbiome of inter and sub-tidal anemone species under increasing pCO2
Source: Sci Rep. 2016 Nov 23;6:37387. doi: 10.1038/srep37387 (PMC5120257; doi:10.1038/srep37387)
Supplement: Supplementary Information [file srep37387-s1.pdf]

Supplementary Information

The stable microbiome of inter and sub-tidal anemone species under increasing pCO_2

Erinn M Muller, Maoz Fine, Kim B. Ritchie

Supplementary Figures

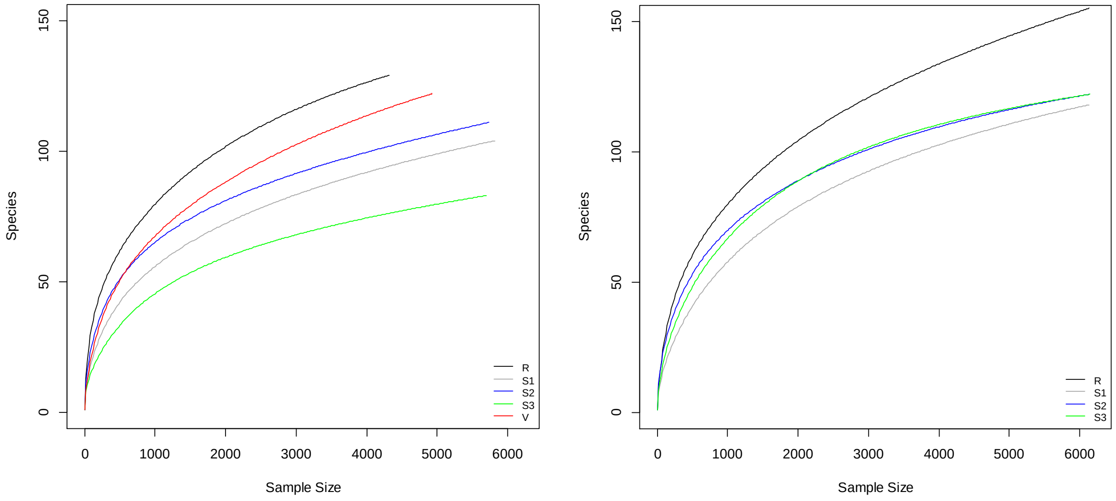

Supplementary Figure 1. Rarefaction curves depicting species richness from bacterial genera data associated with A) *Anemonia viridis* from five sites and B) *Actinia equina* from four sites sampled along a natural pH gradient in Levante Bay, Vulcano. R=reference site, S1=site 1, S2=site 2, S3=site 3, V=vent site.

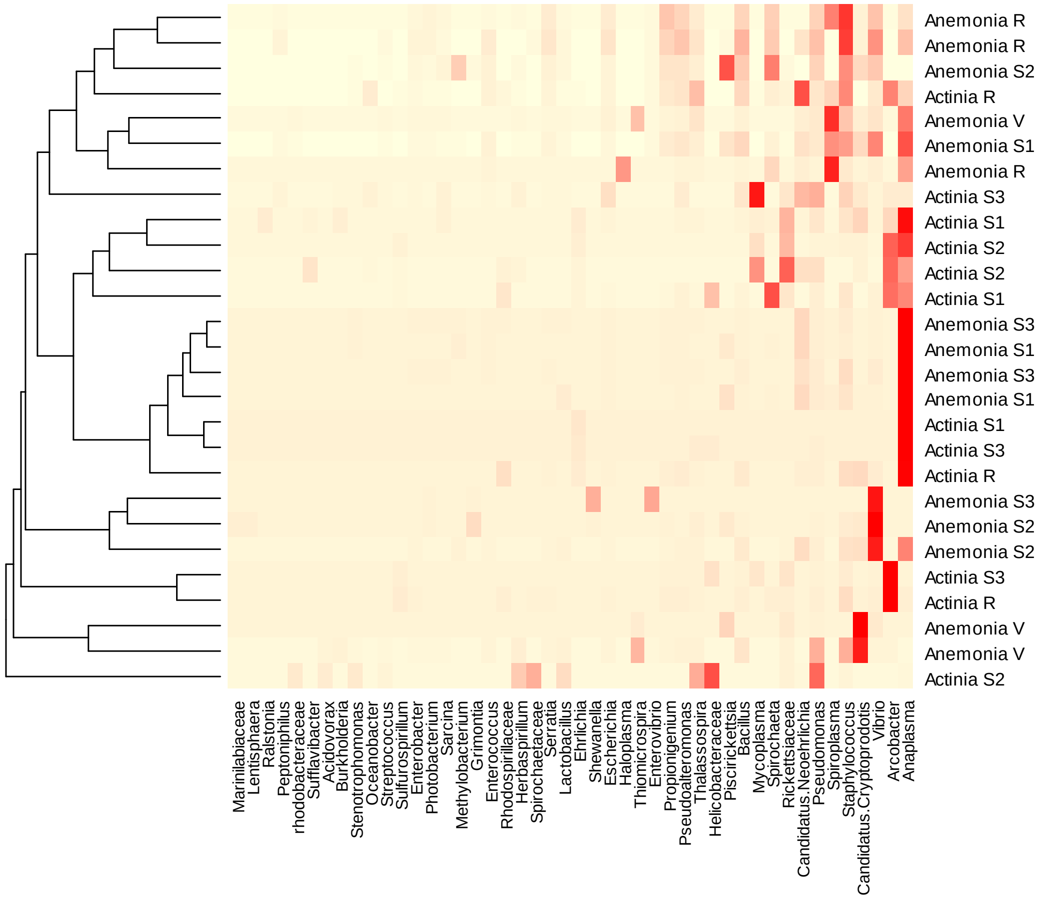

Supplementary Figure 2. Heatmap of the most common genera (found with >1% of the relative contribution to the community) within samples of *Actinia equina* and *Anemonia viridis* collected from Levante Bay, Vulcano, Italy. R=reference site, S1=site 1, S2=site 2, S3=site 3, V=vent site.

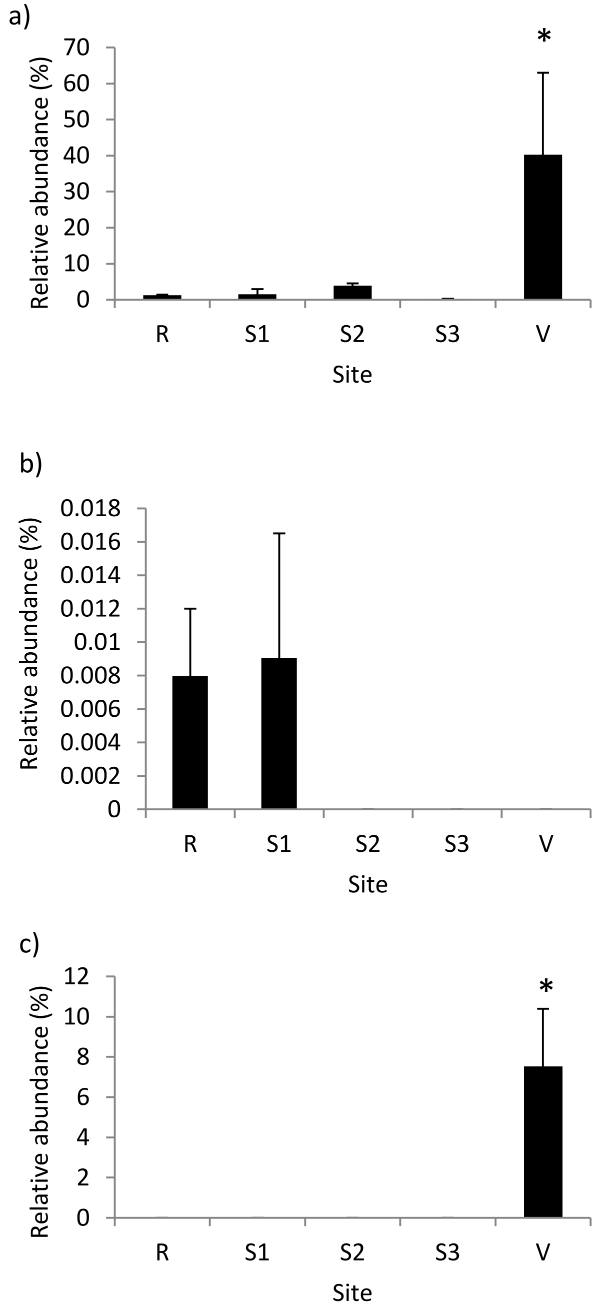

Supplementary Figure 3. Relative abundances of bacterial genera found to significantly differ among sites within samples of *Anemonia viridis* a) *Candidatus Cryptoprodotis* spp., b) *Spiroplasma* spp., and c) *Thiomicrospira* spp. Asterisks represent statistical significance was detected within that site using Dunn's posthoc test with a Bonferroni correction. No posthoc significance was detected among sites within *Spiroplasma* spp.

Supplementary Tables

Supplementary Table 1.

**Identity to reference sequence**	**Identity Designation**
> 97%	Species
Between 97% and 95%	(unclassified Genus)
Between 95% and 90%	(unclassified Family)
Between 90% and 85%	(unclassified order)
Between 85% and 80%	(unclassified class)
Between 80% and 77%	(unclassified phylum)
< 77%	(unknown)

Supplementary Table 2. Coverage of bacterial general from 16S RNA pyrosequencing within each site sampled of *Anemonia viridis* and *Actinia equina*.

Anemone Genus	Site	Coverage estimator
Anemonia	R	0.991
Anemonia	S1	0.991
Anemonia	S2	0.990
Anemonia	S3	0.994
Anemonia	V	0.989
Actinia	R	0.991
Actinia	S1	0.994
Actinia	S2	0.995
Actinia	S3	0.996

Supplementary Table 3. Results of the analysis of variance and Kruskal Wallis tests comparing multiple diversity indices among sites

Anemone Genus	Diversity index	P value
Anemonia	Shannon	0.384
Anemonia	Simpson	0.541
Anemonia	Inverse Simpson	0.541
Anemonia	Pielou’s richness	0.379
Actinia	Shannon	0.659
Actinia	Simpson	0.519
Actinia	Inverse Simpson	0.613
Actinia	Pielou’s richness	0.600

Supplementary Table 4. Results of the pairwise PERMANOVAs comparing the bacterial communities of *Anemonia viridis* among sites.

Site comparison	F value	R2 value	p value
V vs R	1.978	0.331	0.106
V vs S1	2.850	0.416	0.091
V vs S2	1.881	0.320	0.215
V vs S3	1.808	0.311	0.199
S1 vs S2	2.344	0.369	0.227
S1 vs S3	2.380	0.373	0.206
S2 vs S3	1.111	0.217	0.299
R vs S1	2.380	0.373	0.195
R vs S2	1.623	0.289	0.204
R vs S3	1.614	0.287	0.216

Supplementary Table 5. Results of the Kruskal Wallis tests comparing bacterial classes found within *Anemonia viridis* among the five sample sites.

Class	X^2 value	df	p value
Alphaproteobacteria	2.9333	4	0.569
Bacilli	2.0333	4	0.7296
Betaproteobacteria	6.5667	4	0.1606
Clostridia	3.6064	4	0.4619
Epsilonproteobacteria	9.3166	4	0.0537
Fusobacteria	4.5498	4	0.3367
Gammaproteobacteria	4.9667	4	0.2907
Mollicutes	8.961	4	0.0621
Spirochaetes	5.6886	4	0.2236

Supplementary Table 6. Results of the Kruskal Wallis tests comparing bacterial classes found within *Actinia equina* among the four sample sites.

Class	X^2 value	df	p value
Alphaproteobacteria	1.5641	3	0.6676
Bacilli	6.0769	3	0.1079
Betaproteobacteria	0.8974	3	0.826
Clostridia	2.8974	3	0.4077
Epsilonproteobacteria	1.1538	3	0.7641
Fusobacteria	7.158	3	0.067
Gammaproteobacteria	1.2564	3	0.7395
Mollicutes	3.0234	3	0.388
Spirochaetes	2.2121	3	0.5296
